# New Approach to Equitable Intervention Planning to Improve Engagement and Outcomes in a Digital Health Program: Simulation Study

**DOI:** 10.2196/52688

**Published:** 2024-03-15

**Authors:** Jackson A Killian, Manish Jain, Yugang Jia, Jonathan Amar, Erich Huang, Milind Tambe

**Affiliations:** 1 Harvard University Cambridge, MA United States; 2 Verily Life Sciences South San Francisco, CA United States; 3 Google Research Palo Alto, CA United States

**Keywords:** chronic disease, type-2 diabetes, T2D, restless multiarmed bandits, multi-armed bandit, multi-armed bandits, machine learning, resource allocation, digital health, equity

## Abstract

**Background:**

Digital health programs provide individualized support to patients with chronic diseases and their effectiveness is measured by the extent to which patients achieve target individual clinical outcomes and the program’s ability to sustain patient engagement. However, patient dropout and inequitable intervention delivery strategies, which may unintentionally penalize certain patient subgroups, represent challenges to maximizing effectiveness. Therefore, methodologies that optimize the balance between success factors (achievement of target clinical outcomes and sustained engagement) equitably would be desirable, particularly when there are resource constraints.

**Objective:**

Our objectives were to propose a model for digital health program resource management that accounts jointly for the interaction between individual clinical outcomes and patient engagement, ensures equitable allocation as well as allows for capacity planning, and conducts extensive simulations using publicly available data on type 2 diabetes, a chronic disease.

**Methods:**

We propose a restless multiarmed bandit (RMAB) model to plan interventions that jointly optimize long-term engagement and individual clinical outcomes (in this case measured as the achievement of target healthy glucose levels). To mitigate the tendency of RMAB to achieve good aggregate performance by exacerbating disparities between groups, we propose new equitable objectives for RMAB and apply bilevel optimization algorithms to solve them. We formulated a model for the joint evolution of patient engagement and individual clinical outcome trajectory to capture the key dynamics of interest in digital chronic disease management programs.

**Results:**

In simulation exercises, our optimized intervention policies lead to up to 10% more patients reaching healthy glucose levels after 12 months, with a 10% reduction in dropout compared to standard-of-care baselines. Further, our new equitable policies reduce the mean absolute difference of engagement and health outcomes across 6 demographic groups by up to 85% compared to the state-of-the-art.

**Conclusions:**

Planning digital health interventions with individual clinical outcome objectives and long-term engagement dynamics as considerations can be both feasible and effective. We propose using an RMAB sequential decision-making framework, which may offer additional capabilities in capacity planning as well. The integration of an equitable RMAB algorithm further enhances the potential for reaching equitable solutions. This approach provides program designers with the flexibility to switch between different priorities and balance trade-offs across various objectives according to their preferences.

## Introduction

Chronic diseases, while obviously heterogeneous in their physiology, pose a series of common management challenges. One of them is that, by the very nature of these conditions, interventions have to impact multiple aspects of the patient’s daily living to be effective. This scenario is propitious for the implementation of digital health programs (via wearables, mobile apps, or virtual care), such as vida (Vida) and welldoc (Welldoc), that provide patient-centric support between in-clinic visits. These digital health programs may lead to improved clinical outcomes [1-3].

The success of digital health programs, however, hinges on the dynamic balance of several factors. The ultimate metric of success of any program is always the improvement of the individual health outcomes of participants in the program. However, these programs need to sustain participant engagement to be effective [4]. The importance of patients engaging with specific intervention points is clear since only the interventions that patients receive can have an effect. However, sustained engagement over time is a critical success factor in itself, as it can mediate enduring and (potentially) disease-modifying long-term shifts in patients’ attitudes and perceptions about the management of their own health and lifestyle [5]. Yet, attrition and dropout across programs are estimated to be as high as ∼50% [6], representing a major barrier to optimal effectiveness. Moreover, these programs may have capacity limitations (eg, the volume of coaches or health counselors) and need to allocate intervention resources proactively. The options for resource management in digital health programs can vary widely, depending on the metrics and time horizon on which success is measured. In that context, it can be challenging to estimate which approach will be the most effective for a given set of goals.

Our focus is on type 2 diabetes (T2D), which is a representative chronic disease condition. T2D is a high-prevalence, high-burden disease. In the United States, 30 million people are estimated to live with diabetes, the 8th leading cause of mortality [7], and it is estimated to account for over US $300 billion of economic cost [7-9]. The physiologic hallmark of the disease is elevated blood glucose, and success in clinical management is monitored by testing the levels of hemoglobin A_1c_ (HbA_1c_) tests [10]. T2D can lead to organ damage, but it is manageable through medication and lifestyle changes. Our work is based on a digital program that supports patients through a mobile app, virtual coaching (web-based and app-based), and integration of sensor-collected information.

Our digital program of interest contacts patients to maintain engagement and direct and support specific patient actions. Resource investment into those outreach interventions often relies on an intuitive strategy guided by a present clinical state (eg, in this case, giving preference to patients with the highest HbA_1c_). However, such engagement-agnostic strategies may not lead to the best possible health outcomes at the population level, since reactive strategies that only prioritize immediate clinical improvements may do so at the expense of future engagement, reducing the ability to deliver interventions to patients who have dropped out.

Digital programs that consider the joint dynamics of engagement and clinical status may arrive at better determinations about intervention strategies. This is a problem that entails long-term planning usually in resource-constrained settings, therefore it can naturally be cast as a restless multiarmed bandit (RMAB) framework, of the type used for studying resource allocation in the context of stochastic scheduling problems [11]. Recent examples of applying RMABs to health-related problems include computing optimal cancer screening regimens [12], improving maternal health through telehealth [13], and planning hepatitis-C treatment delivery [14].

RMAB frameworks generate sequential resource allocation strategies in pursuit of desired outcomes (in our case it would be optimal health status and engagement) but may be prone to maximizing system-level rewards by sacrificing certain groups to favor the “most promising” ones, hence leading to inequities [15]. In a disease-management context, these (potentially) inequitable policies would translate into disparate outcomes across demographic groups, potentially exacerbating existing systemic inequities in health care [15]. To mitigate this issue, there have been recent studies of fairness in RMAB, in the sense of generating resource allocation strategies with a degree of distributive fairness, where all arms have an opportunity to receive the intervention of interest (in this case resources). Specifically, some works view fairness from the lens of equality, guaranteeing a lower bound of receiving an intervention for all groups [16,17]. Fairness has also been set by modulating risk sensitivity, encoding risk-averseness or risk-prevalence levels to shape the reward functions [18].

In this work, we aimed to develop a resource allocation strategy for a digital health app to support patients with T2D applying an RMAB framework. We intentionally sought to incorporate equity as a desirable feature of our approach, aiming to leverage recent innovations in health care, such as the emergence of digital health, without perpetuating systemic flaws in care delivery, such as societal inequities [15]; moreover, T2D represents an unfortunate example where the presence of systemic inequities continues to have a negative impact in care [19]. We introduce a new solution, equitable RMAB (ERMAB), which requires that allocation policies take affirmative steps to distribute resources in a way that equalizes outcomes across prespecified groups. That is, we focus on fairness through the lens of achieving equitable outcomes in resource allocation. We applied this paradigm to the resource allocation of outreach interventions in our program, evaluating an engagement-health dynamics model and an equitable intervention planning approach via an extensive simulation study using publicly available statistics about digital T2D management. Subsequently, we carried out a Pareto analysis to further study the interplay of engagement-clinical outcome dynamics under different intervention strategies, and perform sensitivity analyses to demonstrate our framework’s robustness across RMAB parameter settings.

## Methods

### Model

#### Overview

Our model needed to simultaneously address the following facets essential to digital health programs: (1) evolution of clinical outcomes per patient, (2) joint engagement-health dynamics per patient, (3) limited observability of clinical outcomes, and (4) limited resource availability.

We model the problem as a restless bandit with n ∈ 1, . . . , N arms representing each patient, discrete per-arm state space S_n_, per-arm action space *A_n_* = {User self-care, Intervention} (equivalently {U, I}), per-arm transition functions *P_n_* defining the probability of arm n transitioning from state s to state s′ given action a, per-arm reward function R_n_ (s) defining the reward for an arm being in state s, time horizon H, and action budget B. For ease of exposition, *S_n_*, *A*_n_, and *R_n_* *(s)* are the same for all arms, so we drop the subscript *n* from these, but our methods apply to the general setting where arms have different state, action, and reward functions. Let *s^t^* be the N-length vector of arm states at time t, indexed as *s^t^_n_*, and let *a^t^* be an N-length 1-hot encoding of the arms that receive interventions from the program in time period *t*. The planner must take actions to maximize their objective, subject to a per-round budget constraint, |*a^t^*|_1_ ≤ *B* ∀t ∈ *1, . . . , H.*

To capture the joint dynamics of engagement and health in digital health programs, we included a dimension for each factor in our state space. For the T2D domain, we also include a dimension for memory, since intervention effects have a delayed impact on clinical outcomes. We represent this 3D state space *S* by a 3-tuple (s_E_, s_C_, and s_M_), where s_E_ captures the arm’s engagement, s_C_ captures the arm’s clinical (ie, health) state, and s_M_ is a 2-length memory vector. All dimensions of the state space are modeled as discrete, where continuous spaces are discretized via threshold rules, described next.

The engagement dimension, s_E_, has 3 states: {*Engaged*, *Maintenance*, and *Dropout*}. A patient is *Engaged* if they received an intervention from the care team and they responded to the team within the app in the current time period. A patient is in the *Maintenance* state if they have produced any interactions within the app, but did not respond to an intervention if it was attempted in the current time period. A patient is in the *Dropout* state if they have not produced any interactions in the app in the current time period and will no longer do so in any future time period (eg, they have deleted the app). These states are chosen to capture the primary high-level engagement dynamics seen in our digital program.

The clinical dimension, s_C_, captures a user’s HbA_1c_ value (via 2 states: {HbA_1c_ < 8, HbA_1c_ ≥ 8}. This threshold was chosen to model the clinical outcome target for app users in publicly available data, that is, reducing their HbA_1c_ below 8. Finally, the memory dimension, s_M_, is a 2-length vector for recording previous values of s_E_, so its entries can take the same values as the s_E_ dimension. The memory serves to implement a 3-month delay between an intervention and its impact on the clinical state. This effect is observed in data and is due to the biological nature of HbA_1c_ progression, that is, it is a summary measure of the body’s blood sugar over the previous 3 months. Let s_Mi_ reference the ith entry of the 0-indexed, 2-length memory vector.

**Figure 1:**
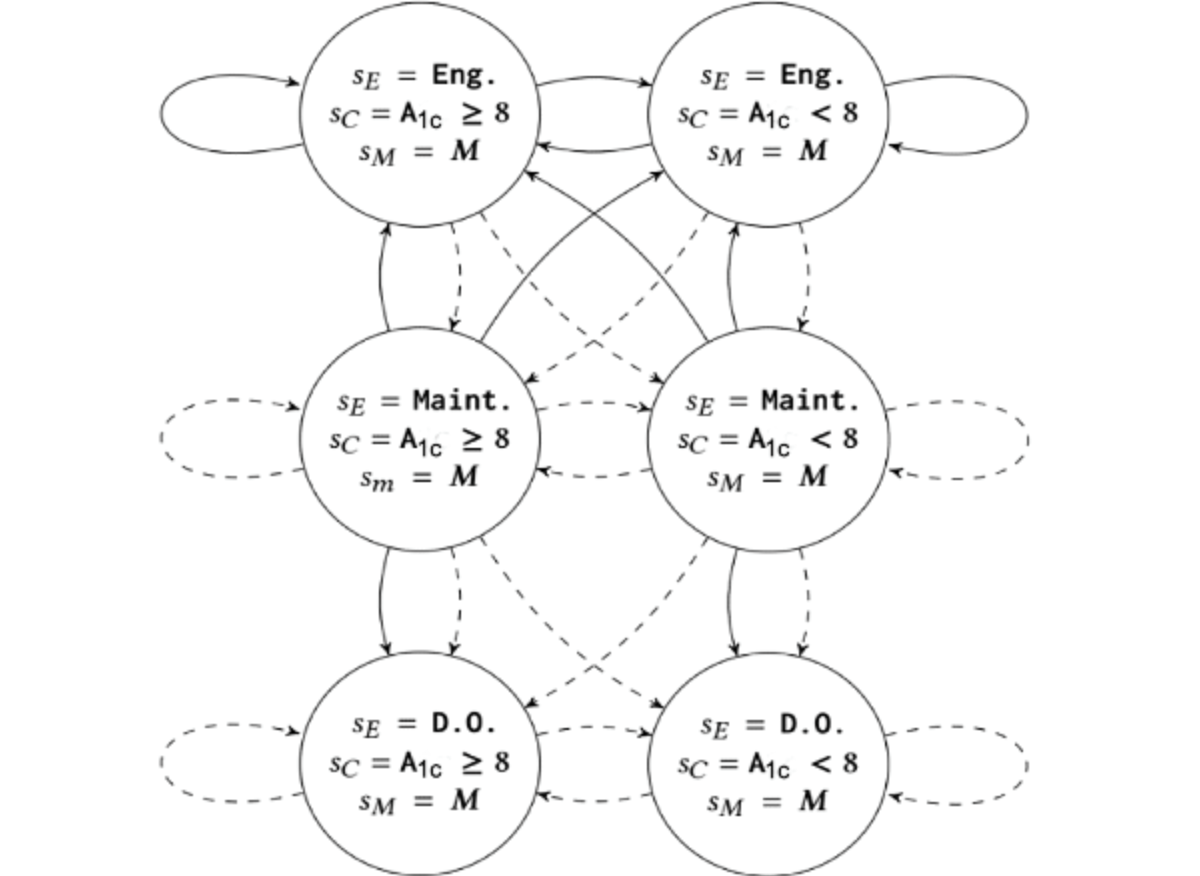
State transition diagram for 1 arm. Bold arrows are transitions when a = intervention and dotted arrows represent transitions when a = user self-care. Eng: engaged; Maint: maintenance.

**Figure 2:**
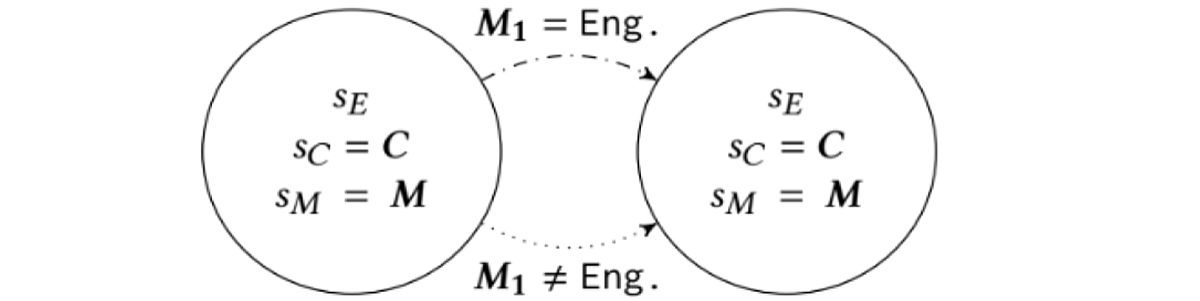
Construction of the delayed intervention effect on clinical state, sC, via zoomed in view of Figure 1. Each transition (arrow) in Figure 1 encodes 2 transitions with different probabilities (the dashed and dotted arrows in this figure), each of which depend on the engagement state of the user 3 months ago, that is, the last entry of the memory state M. Specifically, the probability of transitioning to a better clinical state will be larger if the user was in the engaged state 3 months ago. Eng: engaged.

Transition dynamics are summarized below (Figures 1 and 2).

#### Engagement Dynamics

The engagement model is made up of 4 main effects. First, each patient has their own independent probability of responding to an intervention and transitioning to the *Engaged* state from either the *Engaged* or *Maintenance* states. Second, the probability of a patient responding to an intervention if they were previously in the *Engaged* state is higher than if they were previously in the *Maintenance* state. Third, the probability of a patient transitioning to a *Dropout* state is lower if the patient receives an intervention, than if they do not. Lastly, patients in the *Dropout* state will never respond to an intervention. In summary, this corresponds to 4 open parameters for the engagement dynamics, *p^I^_MtoE_, p^I^_EtoE_, p^I^_MtoD_, and p^U^_MtoD_*, where superscripts, *I* or *U,* denote the action.

#### Clinical Dynamics

There are 2 meaningful clinical dynamics, corresponding to the clinical evolution of patients who did and did not respond to an intervention. Specifically, we assume that patients who received and responded to an intervention (ie, were in the *Engaged* state) will have a higher probability of transitioning to a healthy clinical state than a patient who did not receive or respond to an intervention. In addition, all effects are delayed by 3 months via the memory states as described in the equations below (Figure 2). Note that we assume that HbA_1c_ progression is the same for users who were in the *Maintenance* and *Dropout* states. We show the evolution of the clinical state s′_C_, given the memory state *s_M1_* (ie, clinical state 3 months ago), and the current clinical state *s_C_*, in Table 1. Row 1 of Table 1 represents users who received and responded to an intervention 3 months ago, whereas row 2 represents users who did not receive or respond to an intervention 3 months ago. Note that this requires estimating only 4 parameters for clinical progression, that is, *p^E^_A_1c_≥8,_ p^E^_A_1c_<8_, p^Ē^_A_1c_≥8_, and p^Ē^_A_1c_<8_*, all of which encode the probability of having an HbA_1c_ level less than 8 in 3 months.

**Table 1:** The table shows the evolution of clinical state P (s′C = HbA_1c_ < 8|r, c) where r represents the memory state *s_M1_* and c represents the current clinical state sC.

Evolution of clinical state P (*s′_C_* = HbA_1c_ < 8|*r, c*)	*s′_C_* = HbA_1c_ ≥ 8	*s′_C_* = HbA_1c_ < 8
s_M1_ = Eng^a^	*p^E^_A_1c_≥8_*	*p^E^_A_1c_<8_*
s_M1_ ≠ Eng	*p^Ē^_A_1c_≥8_*	*p^Ē^_A_1c_<8_*

^a^Eng: Engaged.

#### Memory Dynamics

The memory dimension is a sliding window to record the engagement state of the previous 3 months:

P (s′M0 = sE, s′M1 = sM0 | sE, sM0) = 1

Finally, note that the arrows in Figures 1 and 2 represent joint engagement-clinical-memory transition probabilities. These are obtained by multiplying the engagement, clinical, and memory transition rules.

#### Observability

By definition, the engagement state *s_E_*, and thus memory state *s_M_*, are fully observable. However, the clinical state *s_C_* relies on a patient collecting a measurement of their HbA_1c_ in a given time period. We assume that users in the *Engaged* state have fully observable *s_C_*, for example, they will measure their HbA_1c_ upon request from the program, patients in the *Maintenance* state have a partially observable HbA_1c_, for example, they will measure their HbA_1c_ in a given round with probability *q^Obs^_Maint_*, and users in the *Dropout* state have an unobservable HbA_1c_. To handle this partial observability in a computationally scalable way, we convert the partially observable system via a belief-state conversion which allows us to treat the converted system as fully observable [20]. The main benefit is that it allows us to use more efficient optimization tools, at the cost of having a slightly larger state space in the converted system.

#### Rewards

We assign rewards based on the current state of each patient and represent them as *R(s)*. In general, our objective is to jointly boost engagement and clinical state. To capture that objective, we define rewards for each state dimension independently as:



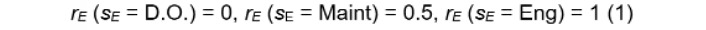





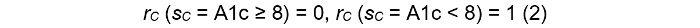



The reward for a patient’s full state is then computed as *R([s_E_, s_C_, s_M_]) = αr_E_(s_E_) + (1 – α) r_C_(s_C_)*.

Thus the parameter *α* represents the relative weight on the engagement reward and it can be tuned based on the planner’s desired objective.

### Equitable Restless Bandit Problem

#### Overview

We model the problem as an RMAB, a framework for finding optimal allocations of constrained resources across many Markov Decision Processes and across time. In this work, we enforce that solutions must also be equitable across *groups* of arms, introducing a new class of ERMAB. Here, we give a brief overview of the ERMAB framework and the equitable objectives considered for our simulation analysis. For full technical background on restless bandits and full derivations of ERMABs and their solutions, please see Killian et al [21].

#### Preliminaries

We consider predefined groups of arms (patients) G, indexed by g. Let M-1(g) be the set of arms in group g. Given a time horizon *H*, a start state s^0^_g_, and per-round budget b_g_, a reward-maximizing allocation policy for a group of arms can be found by computing the value function *V*^0^_g_(*s^0^_g_, b_g_*), where:



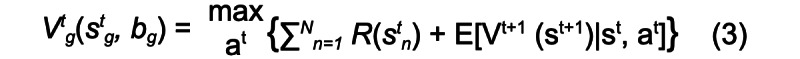





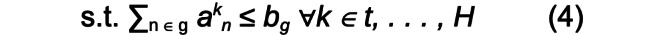



and V^H^_g_(·) = 0. However, solving this exactly is PSPACE-Hard [22], due to the coupling between arms imposed by the budget constraint. Thus, it is more common to work with objectives that relax the budget constraint equation 4 in a Lagrangian fashion, trading some solution quality for computational tractability. Solutions to the relaxed value functions are denoted *L^t^_g_*(*s^t^_g_*, *b_g_*), rather than *V^t^_g_*(*s^t^_g_, b_g_*).

#### Equitable Objectives

In ERMABs, our objective is to both maximize reward and ensure that rewards are distributed equitably across groups of arms. Below, we give 2 objectives for planning such policies.

##### Maximin Reward

Maximin reward (MMR) is a robust objective that maximizes the minimum prospective total reward of any group.



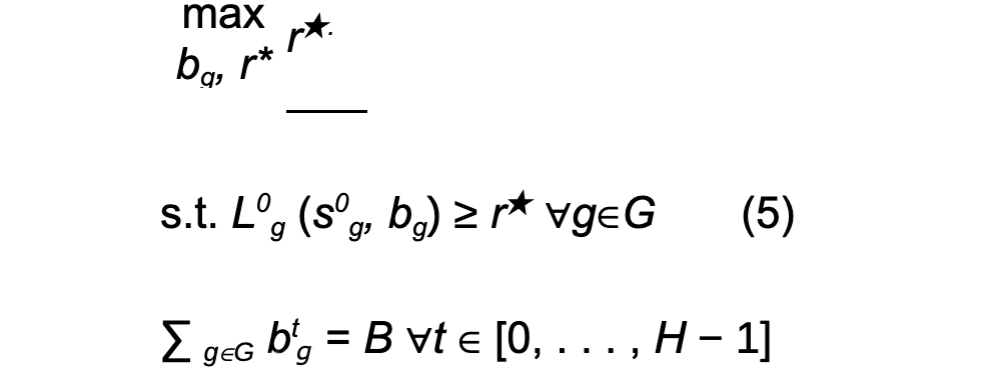



where B is the total per-round budget constraint over all groups. This objective takes a bottom-up approach to equity, ensuring that the groups that are the worst-off are prioritized for resources. However, since the objective focuses only on maximizing the worst case, on some data distributions, it may over-commit resources to a subset of groups with very low potential for improved outcomes, at the expense of potential gains to other groups, which may be undesirable. To account for this, we also consider a second equitable objective that is sensitive to gains across the distribution of groups, while still prioritizing the worst-off.

##### Maximum Nash Welfare



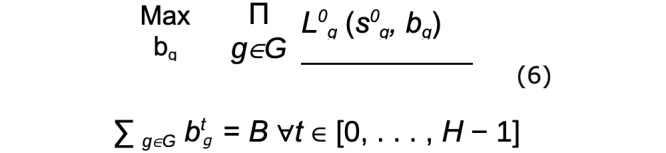



The maximum Nash welfare (MNW) objective gives diminishing returns as the prospective total reward of a group becomes larger. This leads to prioritizing allocations that improve the rewards of all groups more equitably. However, if 1 or a subset of groups have little potential for gains, the allocations will go to the next-worst-off groups which may see some meaningful utility increase from the allocation.

**Figure 3:**
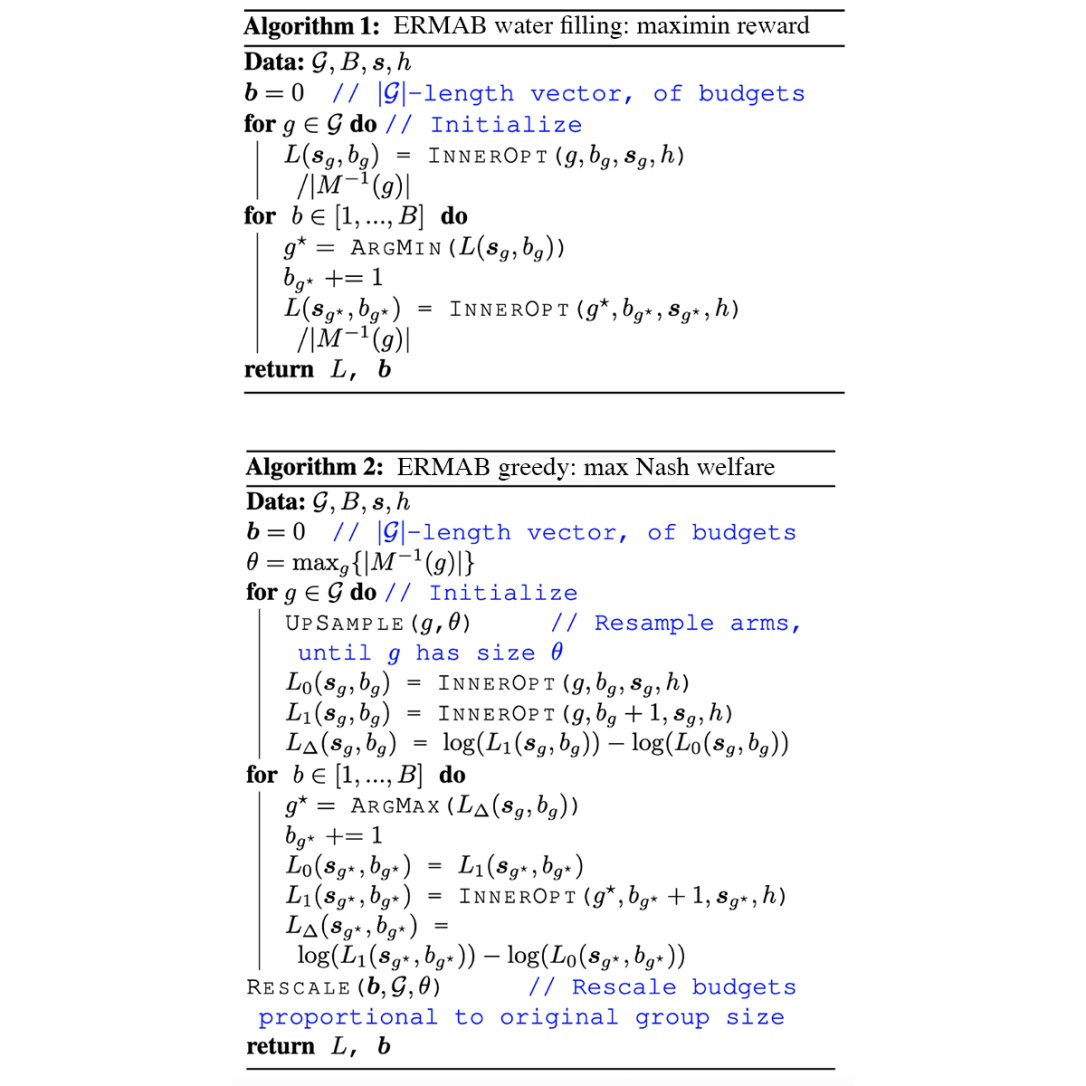
Algorithms. ERMAB: equitable restless multiarmed bandit.

Both objectives represent a natural bilevel optimization problem, where the inner problem solves for the value function *within* 1 group, and the outer problem solves for the equitable distribution of resources *across* groups. To solve equation 5, we use algorithm 1 (Figure 3 [21]), an efficient water filling procedure that incrementally assigns a budget to the group with the smallest long-term value *L*, until the total budget *B* is exhausted. To solve equation 6, we use algorithm 2 (Figure 3 [21]), an efficient greedy approach that incrementally assigns a budget to the group that will see the largest marginal (log) increase in its long-term value *L*, until the total budget *B* is exhausted. The algorithm also includes nuance which corrects for computational biases that occur in the presence of unequal group sizes. To take actions (assign resources) in the simulations, we follow the actions implied by the value functions L^t^_g_(s^t^_g_, b_g_) output by algorithms 1 or 2 (for complete algorithm derivation, with additional proofs and technical detail, see Killian et al [21]).

### Simulation

#### MarketScan Datasource

To derive baseline statistics on clinical evolution, we relied on the widely used Truven Health MarketScan Commercial Database [23], a convenience sample of medical insurance claims from patients who are privately insured in the United States over the years 2018 to 2020, which includes measurements of HbA_1c_. We consider users enrolled for more than 6 months that have T2D only, that is, excluding those with hypertension, depression, heart failure, or cancer. We then group users by age, gender, and starting HbA_1c_ to derive statistics per group on monthly HbA_1c_ change (full details in Multimedia Appendix 1). These provide values of *p^Ē^_A_1c_≥8_* and *p^Ē^_A_1c_<8_* of approximately 7.5% and 0.5%, respectively, with about 1% variation across groups. The MarketScan data set is publicly accessible and provides a reasonable estimate for the background rate of HbA_1c_ change for users not in a specific digital health program, but receiving standard care. It provides a conservative baseline for our experiments.

For the engagement dynamics, statistics on monthly dropout rates by demographic groups from digital health programs are not readily available. Therefore, we use age and gender-based monthly dropout statistics published by the National Diabetes Prevention Program (NDPP) lifestyle change program, primarily made up of in-person meetings [24]. With monthly dropout rates near 10%, this again forms a reasonable conservative baseline for experiments, serving as a proxy for patients’ willingness to engage with T2D-related ongoing behavior change coaching. These statistics populate *p*^U^_MtoD_ in our model, with about 4% variation between groups.

The remaining parameters require estimates from digital health program data which are not readily available publicly. Thus we make the following assumptions to instantiate their values. For *p^E^_A_1c_≥8_* and *p^E^_A_1c_<8_*, that is, the clinical probabilities of patients who received and responded to intervention, the patients in age ranges of aged 30-44, 45-54, and 55-64 years receive 25%, 50%, and 75% boost in their clinical probability of transitioning to HbA_1c_ < 8, respectively. We found that this leads to clinical trajectories in line with 1 published observational study of a digital diabetes management program [25], and included age-based variation to align with variation observed in NDPP’s monthly dropout statistics. For *p*^I^_EtoE_ and p^I^_MtoD_, we assign values of 99% and 3%, respectively, encoding an assumption that patients are more likely to stay in the program if intervened or if already engaged. For *p*^I^_EtoE_, we assign values with a mean of 75%, but with the same group variation as was present in the data for NDPP’s dropout statistics.

Finally, we set the probability of observing the clinical state of a patient in the maintenance state, that is, q^Obs^_Maint_ to 30%, in line with statistics from MarketScan.

#### MMR Counterexample Data

Since MMR objectives are prone to “getting stuck” on unmovable targets, we include a domain to serve as a counterexample that induces this effect. To achieve this, we adopt the probabilities of the MarketScan data, but change the probabilities of 1 group such that interventions are barely effective. Full details are given in the Multimedia Appendix 1.

#### Analyses

Our simulation analyses quantify the extent to which target clinical outcomes are achieved by calculating the numbers and proportions of patients reaching target HbA_1c_ levels (< 8). For all simulation experiments, we started with all patients in the *Engaged* state, with HbA_1c_ ≥ 8, and a memory state of [M, M]. We divided data sets by 3 age ranges (aged 30-44, 45-54, and 55-64 years) and 2 genders (man and woman), creating 6 groups in total. The 6 groups had relative sizes of 0.175, 0.15, 0.2, 0.15, 0.125, and 0.2. To ensure each patient followed a unique behavior profile in simulation, for each patient in a group, we instantiated their transition probabilities by sampling each parameter from a normal distribution using the group value as the mean and σ = 0.05 SD.

Policies were optimized with *α* = .0 unless otherwise noted.

We generated simulation results based on our 2 new equitable policies, MMR and MNW-EG which implemented the MMR and max Nash welfare (with equalized groups) policies, respectively.

We compared simulation results against 2 baselines that served as proxies for how our digital health program of interest, and similar ones, assign intervention resources, that is, based only on the current clinical state. Specifically, allocating interventions randomly each round on patients who are “High Risk,” that is, patients with s_C_ = HbA_1c_ ≥ 8 (termed high-risk random allocation), and a round robin approach which prioritized acting on patients with both s_C_ = HbA_1c_ ≥ 8 *and* with the longest time period without an intervention (termed high-risk round robin allocation).

Additionally, we included a No Action baseline which simulated without assigning any intervention resources, to generate a lower bound of expected outcomes, that is the outcomes observed if individuals were not enrolled in a digital health program, but solely passively seeking care from the traditional primary care system.

We also compared against a state-of-the-art baseline (termed Opt), which assigns resources according to the asymptotically optimal utility-maximizing Whittle index policy [11,26].

### Ethical Considerations

This is a simulation study, without human subject participation. World Medical Association Helsinki Declaration and informed consent guidelines are not applicable.

## Results

### Overview

We evaluated our modeling and algorithmic contributions in simulation environments with data derived from publicly available sources on diabetes progression and health program engagement.

We ran experiments for N ∈ {150, 300, 600} patients, horizon of H = 18 months, for budget values B ∈ {30, 60, 75, 100, 150} and *α* ∈ {0, .25, .50, .75, 1.0}. To simulate gradual patient enrollment over time, a real-world consideration raised by our digital program, 20% of patients are randomly added to the simulation in each of the first 5 months. Final statistics are all reported based on the health state of each patient after their 12th month in the simulation. We use the Gini coefficient [27] concerning each group’s average final reward to measure the equity of each policy applied to each data distribution. Each combination of parameters was run for 50 random seeds, and the results show the average and SE over the seeds.

### Achievement of Target Individual Health Outcomes

**Figure 4:**
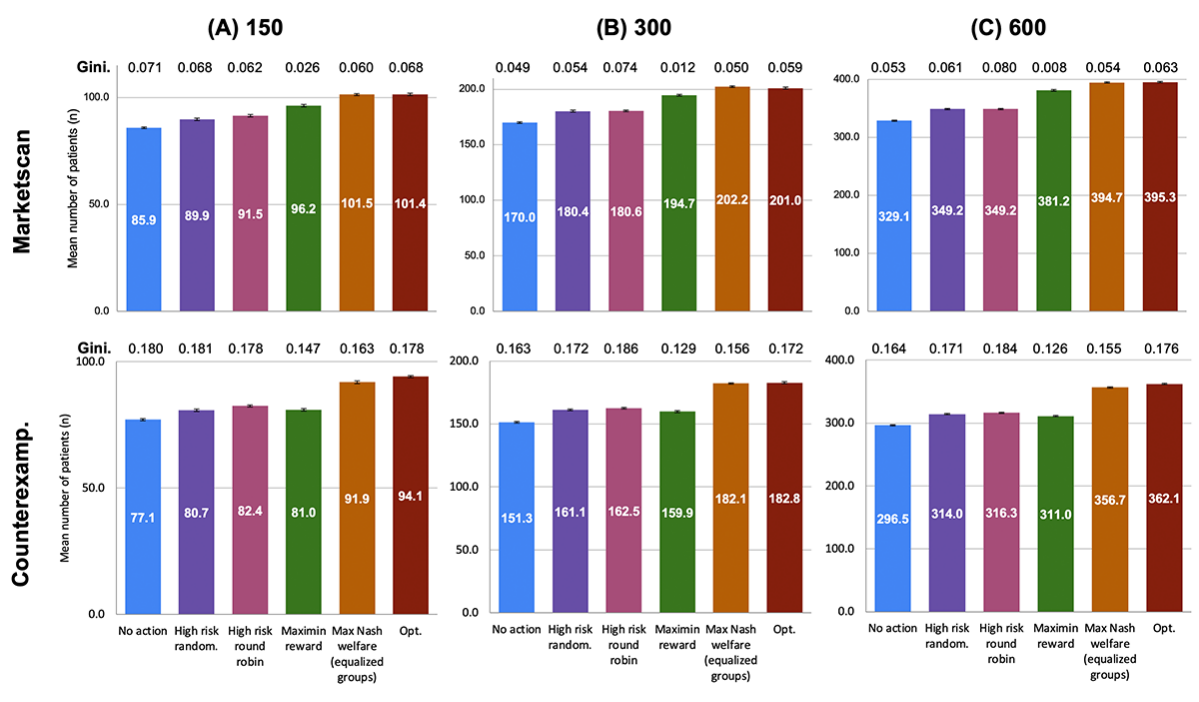
Individual clinical outcomes (average number of patients reaching healthy HbA_1c_ level) with each policy after 12 months, with a monthly intervention budget of B = N 10. Bars show average proportions by policy. Gini coefficient is displayed atop each policy (lower is better). Top: MarketScan. Bottom: MMR-Counterexample. Panels A, B, and C: analyses with N ∈ [150, 300, 600] patients, respectively. Counterexamp: counterexample; Max: maximum; Opt: baseline policy that assigns, based on the optimal utility-maximizing Whittle index policy; random: randomization.

After 12 months, the Opt, MMR, and MNW-EG policies produced better individual clinical outcomes (measured by number of patients reaching healthy HbA_1c_ levels) and engagement than the baselines (Figure 4). The baselines increased the number of users with healthy HbA_1c_ after 12 months by roughly 5%, whereas at the same budget level, assignment policies considering joint clinical-engagement dynamics, that is, the Opt, MMR, and MNW-EG RMAB policies, could double this improvement, up to a further 10% on the MarketScan data set simulation analysis. MMR finds policies nearly 4-times more equitable, for little system-level cost. On the counterexample, MNW-EG avoids the pitfalls of maximin approaches, achieving more equity for little system-level cost.

**Figure 5:**
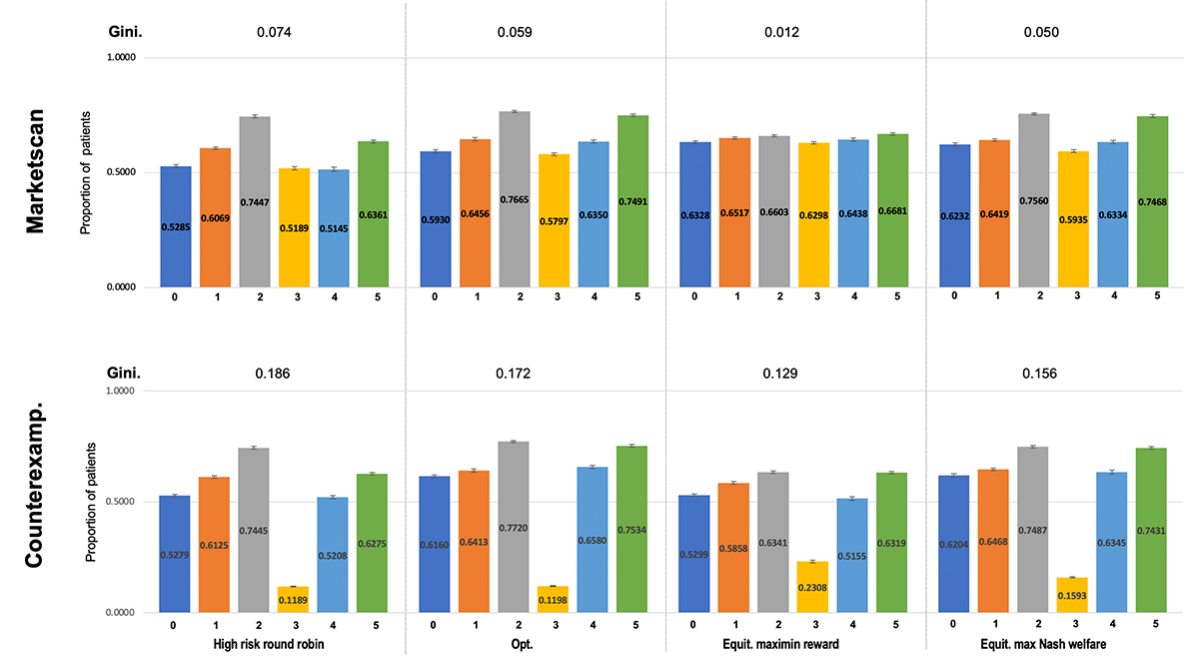
Individual clinical outcomes (proportion of patients reaching healthy HbA_1c_ level) across demographic subgroups. Bars show average proportions by group (0-5) and policy. Gini coefficient is displayed atop each policy. N = 300, B = 30. Top: MarketScan. Bottom: MMR-Counterexample. Counterexamp: counterexample; Equit: equity; Max: maximum; Opt: baseline policy that assigns, based on the optimal utility-maximizing Whittle index policy.

MMR and MNW-EG achieved their lift in the proportion of patients with healthy HbA_1c_ while ensuring greater equity of outcomes across the groups (Figure 5). Specifically, MMR reduced inequity by nearly a factor of 4, at only a slight performance cost. In the counterexample domain (bottom row in the figure), we found that the overly conservative (by design) MMR over-committed resources to improving outcomes of the unmovable group, at the expense of the performance of all other groups. However, in this case, MNW-EG maintained performance as good as Opt, while achieving the most equitable outcomes of any non-MMR policy. We included additional results in the Multimedia Appendix 1 that show analogous results when policies optimize strictly for engagement (ie, *α* = 1.0), conclusions held similarly, although the fair policies were able to achieve even greater improvements to equity over baselines.

### Policy Performance Under Different Preferred Specifications (Pareto Analysis)

**Figure 6:**
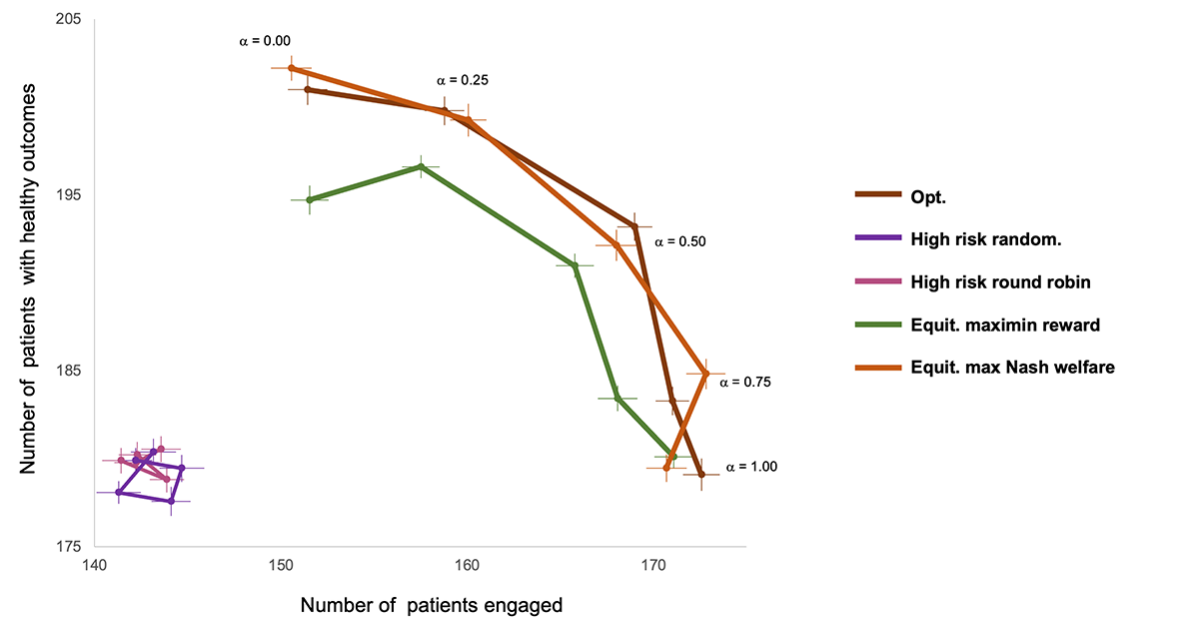
Pareto curve for each policy as *α* varies from 0 to 1, with number of engaged patients after 12 months (ie, in E or M states) on the x-axis and number of patients with healthy clinical outcomes (HbA_1c_ < 8) on the y-axis. The results are shown for the MarketScan data set with N = 300 and B = N 10. Equit: equity; Max: maximum; Opt: baseline policy that assigns, based on the optimal utility-maximizing Whittle index policy; random: randomization.

Pareto analyses (Figure 6) showed that, even with the choice of *α* = 0 (ie, optimizing only for health), MNW-EG and MMR approaches could achieve both improved health and improved engagement compared to clinical-only baselines. Interestingly, for the MarketScan data set, optimizing with *α* = 0.25, that is, a quarter of reward weighted by engagement, could lead to roughly a 10% total reduction in 12-month dropout compared to baselines, while maintaining the 10% boost in 12-month HbA_1c_ targets. We hypothesize that this is due to the “sticky” nature of healthy HbA_1c_ in the MarketScan data set, that is, patients with HbA_1c_ < 8 have a <1% chance of flipping back to HbA_1c_ > 8 in the next month. We give additional results in the Multimedia Appendix 1 for more values of the monthly budget B, and with the Gini index as an axis—the equitable policies remained fairer than Opt across choices of *α* and B.

### Clinical Outcomes According to Resource Allocations: Capacity Planning

**Figure 7:**
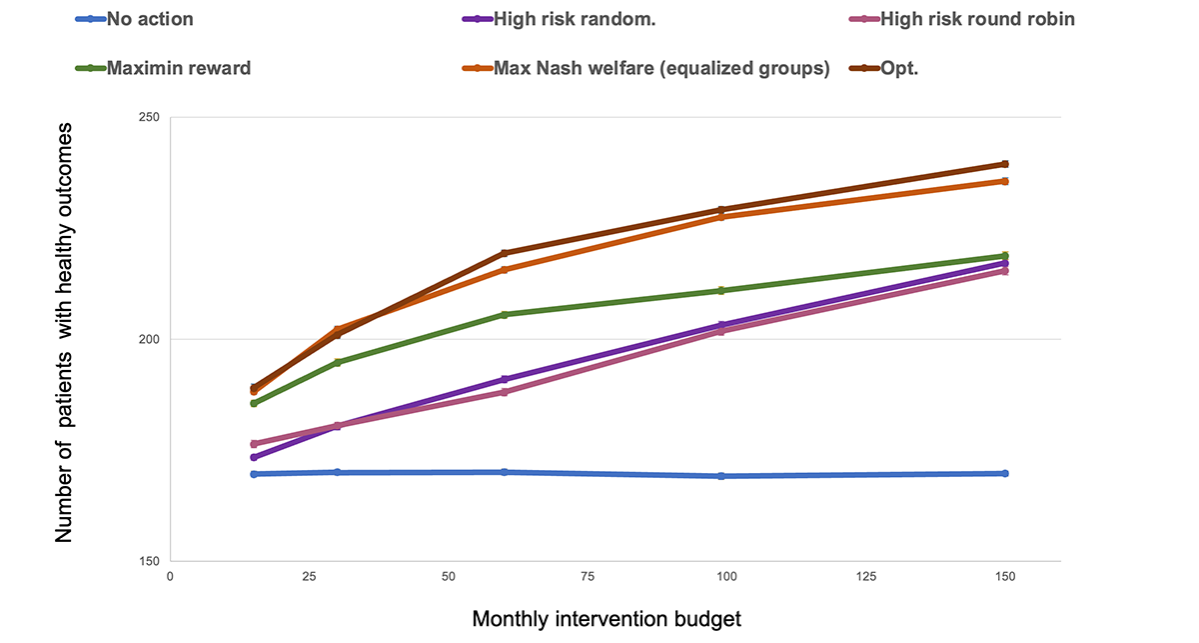
Analysis of individual clinical outcomes according to resource allocation, MarketScan data set, N = 300. In this case, clinical outcomes are measured as the number of patients with healthy outcomes (ie, with HbA_1c_ < 8). Max: maximum; Opt: baseline policy that assigns, based on the optimal utility-maximizing Whittle index policy; random: randomization.

Using the MarketScan data set, we performed analyses to estimate the clinical outcomes resulting from different levels of intervention resource allocations. These analyses demonstrated the capability to perform resource capacity planning for prospective cohorts using our MNW-EG and MMR approaches (Figure 7). For example, if the 12-month target was to reach 200 users with HbA_1c_ < 8, this analysis suggested that roughly 30 intervention resources would be needed if following the Opt policy or MNW-EG policies and 45 resources if following the MMR policies. In contrast, the use of our baseline approaches to reach comparable goals would nearly double the budget, up to 100 monthly intervention resources. Additional results for the counterexample domain, and for *α*-weighted targets, found similar conclusions (Multimedia Appendix 1). These capacity planning plots allowed us to compute the “cost of fairness,” that is, the additional monthly intervention resources required for a more equitable policy to achieve the same total system-level return as the unfair optimal one, by estimating the horizontal difference between where each policy’s line intersects with the target dashed line. In our analysis, the cost of fairness for MNW-EG was negligible, but it was roughly 15 monthly resources for MMR.

## Discussion

### Principal Findings

In this study of a digital health program in T2D, we used a simulation exercise to present a methodological approach to allocate resources in a digital health program with the potential to balance optimization of clinical outcomes, engagement of participants, and distribution of resources in an equitable fashion across participant subgroups. As an example of that potential, in our simplified simulation exercise, optimized intervention policies based on our proposed ERMAB framework led to 10% more patients reaching a healthy clinical outcome (defined by target HbA_1c_ levels) after 12 months, with a 10% reduction in program engagement dropout compared to standard-of-care baselines. Further, these new equitable policies reduced the mean absolute difference (a common equity measure) of engagement and health outcomes across 6 demographic groups by up to 85% compared to the state-of-the-art. We also demonstrated a new capability for a principled capacity planning system. That is, our system allows planners to estimate the required number of intervention resources needed for this digital health program to support a prospective cohort of patients, each with unique support needs and starting state, in reaching target HbA_1c_ levels. While this study was performed in a T2D setting, we believe that the general tenets of our observations may have applications across a spectrum of chronic diseases. Note that, for simulation, we streamlined our modeling approach, with simplified health goals and demographic groups based on age. Therefore, our quantitative results are merely illustrative, but the principles of this approach could be applied and enriched with more sophisticated modeling and other criteria, such as race or ethnicity, geographic location, or other salient sources of existing inequity (as documented in diabetes care [19]), when information about those factors is available.

### Comparison to Other Work

This work is related to a wide literature on using machine learning to make predictions in support of the delivery of digital health. Examples include predicting mood and depression [31], predicting medication adherence [32], ranking the efficacy of smoking cessation messages [33], and predicting heart arrhythmias from smart watches [34]. There are, however, several elements that contrast this study from others. While other works make predictions about the current or future states of a patient’s health, they do not offer tools for planning the allocation of resources. Our work focuses on building up the algorithmic tools required for the long-term planning of allocations of limited resources in ways that will benefit the digital health system as a whole.

This study is also the first to formulate an RMAB model of digital health which has the novel characteristic of a multidimensional state space that encodes the joint dynamics of engagement and clinical health, giving the problem a relevant new structure, but increasing the computational complexity over previous domains.

Furthermore, we had equity-focused objectives, which viewed fairness through the lens of taking affirmative steps toward equitable outcomes. Overall considerations of equity in digital health are an underdeveloped area of study; prior or ongoing studies are still trying to measure the inequality problem in digital health in terms of usability, access, or feedback opportunities [35-39]. Most results show that societal inequalities at large have a reflection in the field of digital health, compounded by the issue of uneven technical access. These findings lend more urgency to the development of optimizing strategies that tackle the problem of inequality intentionally and proactively. Our work is novel in that it proposes to formulate digital health programs to achieve outcome-based fairness. To our knowledge, this is the first study of its kind leveraging restless bandits and the first to give a principled framework for solving the problem of equitable outcomes with guarantees, in contrast to previous work on probabilistic fairness, which merely guaranteed each arm a lower bound of being considered for an intervention [16,17].

### Specific Strengths

In addition, we demonstrated a key new capability of interest to digital health program administrators, namely the ability to perform resource capacity planning for prospective cohorts. This feature allows, for instance, to answer the question of whether the digital health program needs the same number of intervention resources to support a cohort of people aged 55-64 years from a particular region as it does to support a cohort of people aged 35-44 years from a different location. Given estimates of each cohort’s clinical and engagement behaviors derived from historical data, one can simulate their preferred intervention policies to understand how many resources are needed to reasonably expect each cohort to reach their clinical goals. Capacity planning analysis, coupled with group-level evaluations of policy equity should allow planners to make principled decisions about resource needs for different populations.

### Limitations

We acknowledge that this study also has limitations. As reported in this paper, we have only conducted simulation exercises with the analytical framework that we are proposing. We found the simulated results encouraging regarding the potential of our approach to achieve the objectives of allocating digital health program resources in a manner that is effective for reaching individual target clinical outcomes, and for maintaining patient engagement and population-level equitable care delivery throughout the process. However, further research applying this ERMAB framework in a real-world context is warranted to confirm the upside potential shown in simulations. In addition, our T2D model is simplified and we used claims data for our simulations; claims have limitations as sources when inquiries go beyond information directly related to medical procedures, thus we opted for a simplified strategy accordingly. First, we are modeling a binary distinction for HbA_1c_ outcomes (< 8 or ≥ 8); while there is precedent for this approach, this simplification is still a limitation of the model. This cutoff point may not be optimal for all patients [40]. Second, the model does not consider comorbidities, which are highly relevant in diabetes, and chronic conditions in general, and could have meaningful effects on outputs, particularly the individual health outcomes. However, this model can be expanded with more granularity, as long as it can learn additional parameters from more sophisticated real-world data sets. These considerations (more individualized HbA_1c_ outcomes, comorbidities, and relevant subcohorts to the investigation of inequity) will all be important for future research based on other sources (such as electronic health records or clinical registries), to determine to which extent increasing complexity in the desired outcomes may affect the model’s performance, and the practical implementation of the results.

### Conclusions

In conclusion, our work showed the potential feasibility of planning interventions in digital health attending to several important factors in today’s societal environment and resource-constrained systems. Our approach to intervention planning accounts not only for individual clinical outcome objectives but also for long-term participant engagement dynamics, using an RMAB sequential decision-making framework. We were able to simulate more equitable policies that could jointly improve engagement as well as clinical outcomes and demonstrated how the RMAB simulation framework could also provide key new capabilities in capacity planning, and objectively analyze how to trade-off between different program outcomes. Finally, we make a key new algorithmic contribution by introducing ERMABs and designing an efficient and fair approach for reaching population-level equitable solutions. We hope that ERMABs will add to the arsenal of tools available to practitioners addressing resource allocation problems in ethically sensitive domains.

## Appendix

Multimedia Appendix 1 Additional description of methods.

## Abbreviations

ERMAB: equitable restless multiarmed bandit
HbA_1c_: hemoglobin A_1c_
MMR: maximin reward
MNW: maximum Nash welfare
NDPP: National Diabetes Prevention Program
Opt: baseline policy that assigns, based on the optimal utility-maximizing Whittle index policy
RMAB: restless multiarmed bandit
T2D: type 2 diabetes
