# Supplementary material for: New Approach to Equitable Intervention Planning to Improve Engagement and Outcomes in a Digital Health Program: Simulation Study
Source: JMIR Diabetes. 2024 Mar 15;9:e52688. doi: 10.2196/52688 (PMC10980993; doi:10.2196/52688)
Supplement: Multimedia Appendix 1 [file diabetes_v9i1e52688_app1.pdf]

Multimedia Appendix 1:

A New Approach to Equitable Intervention Planning to Improve Engagement and Outcomes in a Digital Health Program: Simulation Study

Jackson A. Killian[1] **Manish Jain**[2] **Yugang Jia**[3] **Jonathan Amar**[3] **Erich Huang**[3] **Milind Tambe**[1,2]

[1]Computer Science, Harvard University
[2]Google Research
[3]Verily Life Sciences

A1 DATASET DETAILS

A1.1 THE MARKETSCAN DATASET.

We consider users enrolled for more than 6 months that have T2D only, i.e., excluding those with hypertension, depression, heart failure, or cancer. We filter to patients who had at least two measurements separated by more than 28 days, which comprise 35k patients. We then compute each patient's monthly A1c change based on the difference between their A1c readings separated the furthest in time, to reduce noise inherent in more adjacent readings. We then group patients by starting A1c (greater or less than 8) and compute the average and standard deviation of starting A1c and monthly A1c change per group. Finally, we convert those statistics to monthly probabilities $p^{\overline{\mathbb{E}}}_{A1c\geq8}$ and $p^{\overline{\mathbb{E}}}_{A1c<8}$ by computing the number of patients who would reach or remain at an A1c of 8 or less assuming a normal distribution for (1) starting A1c per group and (2) monthly A1c change per group. These provide values of $p^{\overline{\mathbb{E}}}_{A1c\geq8}$ and $p^{\overline{\mathbb{E}}}_{A1c<8}$ of approximately 7.5% and 99.5%, respectively, with total variation across groups about 1 percentage point. The final table of parameters is given in Table A1.

A1.2 MMR COUNTEREXAMPLE DATASET.

To create this altered dataset, we changed the parameters of group from the MarketScan dataset such that interventions would not be effective. This causes MMR to over-commit resources that group at the expense of system-level gains. See Table A2.

A2 ADDITIONAL EXPERIMENTAL RESULTS

Figs. A1 and A9 and show results total and group-distributed rewards with all strategies optimized for and measured by engagement ($\alpha = 1.0$). Equitable policies again achieve similar reward as unfair Opt, but with Gini coefficients that are multiple factors improved. MMR performs better than for the health-focused case.

Figs. A2 and A3 show Pareto curves for additional values of the budget B, with similar conclusions as the main text. Figs. A4 and A5 show Pareto curves with Gini coefficient on the y-axis and health and engagement on the x-axis, respectively. Lower to the right is better. The equitable policies maintain their superior fairness and comparable system-level performance across various values of α.

Finally, Figs. A6, A7, and A8 show capacity planning plots for Marketscan with $\alpha = 0.25$, MMR-Counterexample with $\alpha = 0$ and MMR-Counterexample with $\alpha = 0.25$, respectively, as further proof of concept on other data distributions.

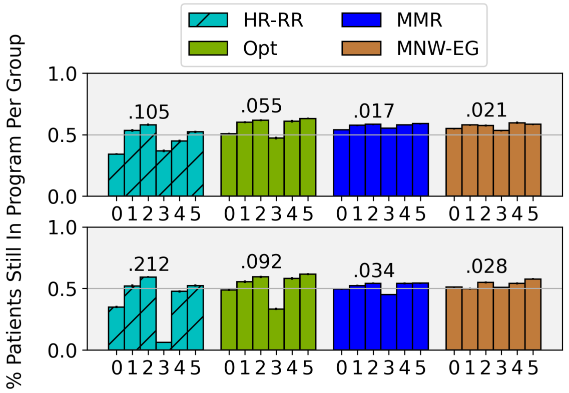

Figure A1: Top: MarketScan. Bottom: MMR-Counterexample. Bars show average final number of patients in engaged or maintenance state by group (0–5) and policy. Gini coefficient is displayed atop each policy. $N = 300, B = 30$. Policies were optimized with $\alpha = 1.0$

Table A1: MarketScan experiment parameters.

Group	p^{I}_{MtoE}	p^{I}_{MtoD}	p^{I}_{EtoE}	p^{U}_{MtoD}	$p^{\overline{E}}_{A1c\geq8}$	$p^{\overline{E}}_{A1c<8}$	$p^{E}_{A1c\geq8}$	$p^{E}_{A1c<8}$	frac	sex	age
0	0.560	0.03	0.99	0.122	0.071	0.992	0.089	0.994	0.175	1	30-44
1	0.783	0.03	0.99	0.093	0.074	0.990	0.111	0.995	0.150	1	45-54
2	0.907	0.03	0.99	0.077	0.080	0.993	0.140	0.998	0.200	1	55-64
3	0.560	0.03	0.99	0.122	0.069	0.992	0.087	0.994	0.150	2	30-44
4	0.783	0.03	0.99	0.093	0.070	0.993	0.104	0.996	0.125	2	45-54
5	0.907	0.03	0.99	0.077	0.085	0.995	0.148	0.999	0.200	2	55-64

Table A2: MMR-Counterexample experiment parameters. Group 3 modified to respond poorly to intervention.

Group	p^{I}_{MtoE}	p^{I}_{MtoD}	p^{I}_{EtoE}	p^{U}_{MtoD}	$p^{\overline{E}}_{A1c\geq8}$	$p^{\overline{E}}_{A1c<8}$	$p^{E}_{A1c\geq8}$	$p^{E}_{A1c<8}$	frac	sex	age
0	0.560	0.03	0.99	0.122	0.071	0.992	0.089	0.994	0.175	1	30-44
1	0.783	0.03	0.99	0.093	0.074	0.990	0.111	0.995	0.150	1	45-54
2	0.907	0.03	0.99	0.077	0.080	0.993	0.140	0.998	0.200	1	55-64
**3**	**0.500**	**0.03**	**0.99**	**0.300**	**0.020**	**0.800**	**0.025**	**0.850**	**0.150**	2	30-44
4	0.783	0.03	0.99	0.093	0.070	0.993	0.104	0.996	0.125	2	45-54
5	0.907	0.03	0.99	0.077	0.085	0.995	0.148	0.999	0.200	2	55-64

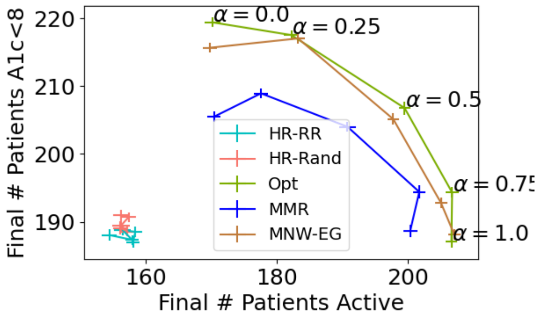

Figure A2: Pareto curve showing engagement vs. health, with $N = 300$ and $B = 60$, MarketScan dataset.

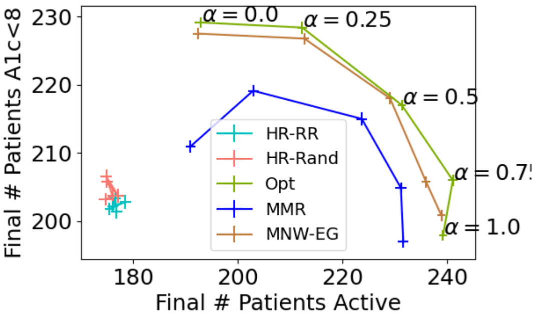

Figure A3: Pareto curve showing engagement vs. health, with $N = 300$ and $B = 100$, MarketScan dataset.

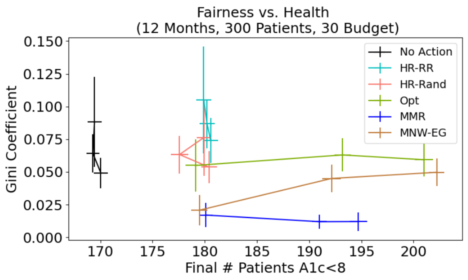

Figure A4: Pareto curve showing Gini Coefficient vs. health for various alpha, with $N = 300$ and $B = 30$, MarketScan dataset. Low and right is better.

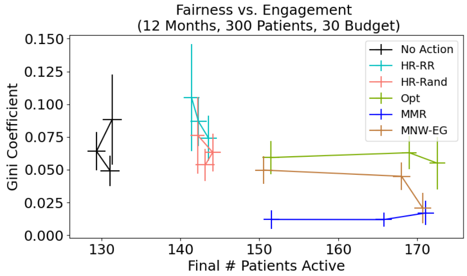

Figure A5: Pareto curve showing Gini Coefficient vs. engagement for various alpha, with $N = 300$ and $B = 30$, MarketScan dataset. Low and right is better.

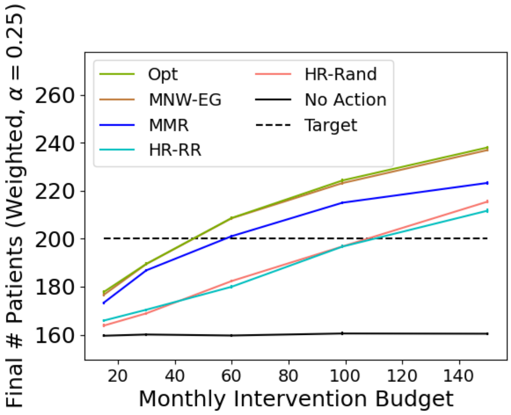

Figure A6: Capacity planning for the MarketScan dataset with $\alpha = 0.25$, $N = 300$

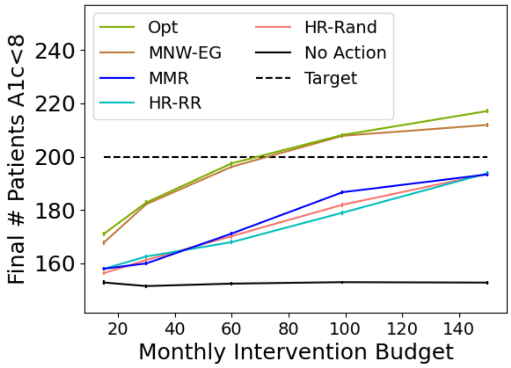

Figure A7: Capacity planning for the MMR-Counterexample dataset with $\alpha = 0.0$, $N = 300$

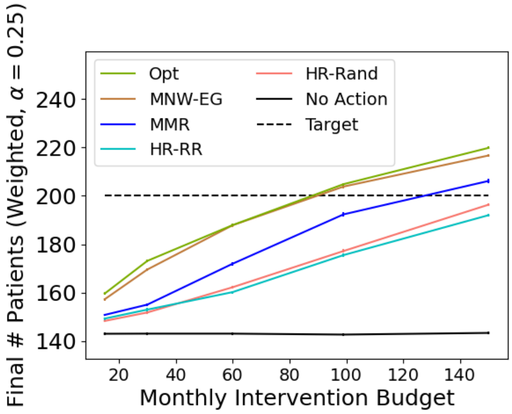

Figure A8: Capacity planning for the MMR-Counterexample dataset with $\alpha = 0.25$, $N = 300$

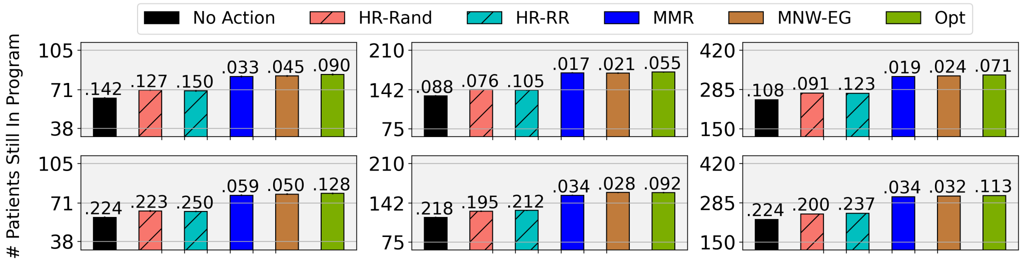

Figure A9: Top: MarketScan dataset. Bottom: MMR-Counterexample dataset. Left, middle, and right columns have $N = [150, 300, 600]$ arms, respectively, with $B = N/10$. Planning with $\alpha = 1.0$ Bar height shows the number of patients still engaged or in maintenance state at the end of each patient's 12th month. The number on top of the bar is the Gini coefficient (smaller is better).
